# Genetic Surveillance Reveals Differential Evolutionary Dynamic of *Anopheles gambiae* Under Contrasting Insecticidal Tools Used in Malaria Control

**DOI:** 10.1111/mec.70284

**Published:** 2026-03-03

**Authors:** Harun N. Njoroge, Lilian Namuli, Sanjay C. Nagi, Anastasia Hernandez‐Koutoucheva, Daniel P. McDermott, Erin Knight, Samuel Gonahasa, Amy Lynd, Ambrose Oruni, Catherine Maiteki‐Sebuguzi, Jimmy Opigo, Adoke Yeka, Agaba Katureebe, Mary Kyohere, Moses R. Kamya, Grant Dorsey, Janet Hemingway, Sarah G. Staedke, Chris Clarkson, Alistair Miles, Eric R. Lucas, Martin J. Donnelly

**Affiliations:** ^1^ Liverpool School of Tropical Medicine Liverpool UK; ^2^ Infectious Diseases Research Collaboration Kampala Uganda; ^3^ National Malaria Elimination Division, Ministry of Health Kampala Uganda; ^4^ Makerere University College of Health Sciences Kampala Uganda; ^5^ University of California, San Francisco San Francisco California USA; ^6^ Ellison Institute of Technology Oxford UK

## Abstract

Malaria, a febrile disease caused by the *Plasmodium* parasites and transmitted by mosquitoes, is a leading cause of mortality in children under 5 in endemic countries. The widespread deployment of insecticide‐treated bed nets (ITNs) has significantly reduced malaria transmission, but rising levels of insecticide resistance threaten to halt the progress. Monitoring insecticide resistance is vital for effective vector control, particularly when deploying new tools. Understanding mosquito population responses to these interventions is crucial for guiding control programmes in making informed decisions about the selection, timing and geographic deployment of tools. This genomic study investigates the demographic and evolutionary consequences on the malaria vector *Anopheles gambiae* of deploying standard ITNs (containing only pyrethroids) and pyrethroid‐PBO nets (containing pyrethroids plus the synergist piperonyl butoxide) during a clinical trial in Uganda. Despite substantial reductions in indoor mosquito densities in the clinical trial, estimates of nucleotide diversity (π) and linkage disequilibrium revealed no significant decline in effective population size, reflecting continued large population size even after effective control. Marked allele frequency shifts at resistance‐associated loci indicated strong selection pressures driven by the interventions, with distinct selective dynamics between the two net types, highlighting alternative pyrethroid detoxification pathways in the presence of PBO. A duplication in the *Cyp9k1* gene significantly increased in frequency in populations exposed to pyrethroid‐only nets but decreased in populations exposed to PBO‐treated nets, suggesting that selection for over‐expression of this gene is removed when this resistance mechanism is impacted by PBO. An alternative potential detoxification mechanism was selected within a region of the 2La chromosomal inversion on chromosome 2 L, which encompasses the UDP‐glucose 6‐dehydrogenase gene. This variant consistently increased in frequency when exposed to PBO‐treated nets. Additionally, pyrethroid‐only nets selected for a novel locus on the X chromosome containing the diacylglycerol kinase gene, which is potentially linked to behavioural adaptations through its role in neurotransmission modulation. Our findings underscore the importance of genomic surveillance in vector control, revealing distinct evolutionary dynamics of insecticide resistance mechanisms in the presence of PBO. While ITNs remain effective, the persistence and evolution of resistance‐associated alleles highlight the need for adaptive and dynamic resistance management strategies. By integrating high‐resolution genomic data with epidemiological and entomological monitoring, this study offers actionable insights to sustain malaria control efforts amid the ongoing challenge of insecticide resistance.

## 1 Introduction

Malaria accounts for over half a million deaths each year, predominantly in sub‐Saharan Africa (WHO 2024). Vector control methods using insecticides, particularly insecticide‐treated bed nets (ITNs) and indoor residual spraying, are the main tools used in malaria control (Bhatt et al. 2015; Wilson et al. 2020). However, the emergence of insecticide resistance in *Anopheles* mosquitoes, the primary malaria vectors, necessitates innovative and sustainable strategies to achieve malaria elimination (Cotter et al. 2013; Mnzava et al. 2015). Effective malaria control requires rigorous evaluation of vector control tools through controlled trials and an understanding of how mosquito populations respond to them (Wilson et al. 2020).

Traditionally, malaria vector control has been monitored using entomological endpoints, including vector density, sporozoite infectivity rates and entomological inoculation rates (EIR) (Morales‐Pérezid et al. 2020; Traore et al. 2020). While these indicators remain the industry standards, they offer limited insights into the mechanisms underlying control effectiveness and are subject to biases with mosquito trapping techniques, environmental conditions and technician proficiency, complicating comparisons between interventions (Rohani et al. 2016; Swai et al. 2023; Degefa et al. 2024).

Advances in genetic monitoring, informed by fields such as conservation genetics and pest management, provide opportunities to assess vector population dynamics, insecticide resistance and intervention effectiveness with greater precision (Neafsey et al. 2021; Schmidt et al. 2021). Integrating genomic surveillance with traditional epidemiological indicators offers a promising approach to understanding vector population responses to control measures (Fouet et al. 2018). For instance, population genetics metrics such as F_ST_, heterozygosity, nucleotide diversity and effective population size (N_e_) have been employed to assess the impact of ITNs on vector abundance (Cartaxo et al. 2011; Athrey et al. 2012; O'Loughlin et al. 2016; Huang et al. 2023).

Genetic surveillance approaches can augment traditional phenotypic assays for insecticide resistance, which involve direct insecticide exposure. These assays are influenced by environmental factors, mosquito physiology and technician variability, limiting replicability (Praulins et al. 2024). Molecular diagnostics of targeted loci address some of these challenges, but while many markers are available for surveillance of insecticide resistance, they do not at present explain the full variation in resistance (Ranson et al. 2000; Jones et al. 2012; Weetman et al. 2018; Njoroge et al. 2022; Lucas et al. 2023), and are limited to discovering mutations at known loci.

Pragmatic clinical trials leverage real‐world control implementations, allowing efficient use of resources and testing of products in real applications. In this study, we use whole‐genome sequencing (WGS) of *An. gambiae s.s*. collected during a large pragmatic cluster‐randomised trial embedded within a national bed net distribution campaign in Uganda between 2017 and 2019, the LLINEUP trial (Staedke et al. 2020). The trial evaluated the effectiveness of standard pyrethroid‐only long‐lasting insecticide‐treated bed nets (LLINs) against LLINs containing a pyrethroid + piperonyl butoxide (PBO) across 104 health sub‐districts of Uganda. In Uganda, metabolic pyrethroid resistance mediated by cytochrome P450 enzymes is widespread and linked to a rapidly spreading resistance haplotype (Njoroge et al. 2022). Because the action of P450s is inhibited by PBO, the PBO + pyrethroid bed nets are expected to overcome the metabolic resistance, making them more effective. This was reflected in the epidemiological and entomological measurements, which revealed a parasite prevalence ratio of 0.86 (0.74–1.00) and a vector density ratio of 0.38 (0.29–0.49) in the PBO + pyrethroid arm relative to the pyrethroid‐only arm (Maiteki‐Sebuguzi et al. 2023). Given the contrasting insecticidal pressure experienced by the mosquito population in the two arms, the trial offers an unprecedented opportunity to investigate the effects of large‐scale vector interventions on the evolution and genetic makeup of a targeted vector population, particularly to elucidate the evolutionary impact of PBO on cytochrome P450‐mediated adaptive mechanisms.

We hypothesised that (H_1_) the WGS data would reveal decreases in measures of vector effective population size across the entire study, (H_2_) reductions in N_e_ would be greater in the PBO arm compared to the pyrethroid‐only arm, (H_3_) adaptive alleles would change in frequency across the study and (H_4_) distinct changes in frequencies of adaptive alleles would occur in the different arms, in particular for cytochrome P450 genes.

## 2 Methods

### 2.1 LLIN Evaluation in Uganda Project (LLINEUP): Study Design, Entomological Sampling and Sample Processing

#### 2.1.1 Study Design

The Long‐Lasting Insecticidal Net Evaluation in Uganda Project (LLINEUP) was designed to assess the impact of LLINs, with and without PBO, on malaria indicators in Uganda. Conducted as a cluster‐randomised trial embedded within a national LLIN distribution campaign (2017–2018), the study included two arms: one receiving standard pyrethroid nets and the other receiving PBO‐pyrethroid impregnated nets.

A total of 104 health sub‐districts (clusters) in Eastern and Western Uganda participated in the trial. Of these, 52 clusters were allocated PBO LLINs (PermaNet 3.0 and Olyset Plus), while 52 clusters received conventional LLINs (PermaNet 2.0 and Olyset Net). The primary outcome was malaria parasite prevalence in children aged 2–10 years, measured via microscopy. Secondary outcomes included anaemia prevalence, vector density, LLIN coverage, ownership and usage. Details of the trial design, including sample size calculations and randomization methods, are available in previous publications (Staedke et al. 2019; Maiteki‐Sebuguzi et al. 2023).

#### 2.1.2 Entomological Sampling

Entomological surveillance was conducted to assess vector density, monitor insecticide resistance and collect genomic data. Sampling was performed in all 104 clusters at baseline (prior to LLIN distribution), at 6‐, 12‐ and 18 months but in 90 clusters at 25 months post‐distribution. In each cluster, 10 households were randomly selected for indoor resting mosquito collection using Prokopack aspirators. Female *Anopheles* mosquitoes were identified morphologically, preserved in silica gel and shipped to the Liverpool School of Tropical Medicine (LSTM) for further processing. Detailed sampling protocols are described elsewhere (Lynd et al. 2024). Previous analysis of these data showed that catch numbers of female anophelines declined after bed net deployment and more so in pyrethroid + PBO than pyrethroid only nets (Table S1a Maiteki‐Sebuguzi et al. 2023; Staedke et al. 2020).

#### 2.1.3 Sample Processing

DNA was extracted from individual *Anopheles* mosquitoes using Nexttec extraction kits (Biotechnologie GmbH). Species identification was performed using a species‐specific PCR assay targeting a SINE insertion site, distinguishing *An. gambiae s.s*., *An. arabiensis* and *An. coluzzii* based on melt curve analysis (Chabi et al. 2019). This study focuses on *An. gambiae*, Uganda's primary malaria vector at the time (Lynd et al. 2019). A subset of *An. gambiae* samples within each entomological collection round and considering a good coverage across the health sub‐districts were whole genome sequenced. Sequencing, alignment, variant calling and phasing were carried out by the Malariagen Vector Observatory (The Anopheles gambiae 1000 Genomes Consortium 2017) using the *An. gambiae* 1000 genomes project pipeline that is described in the GitHub repository https://github.com/malariagen/pipelines/blob/c7210d93628aaa31f26baa88a92e10322368b78e/docs/specs/short‐read‐alignment‐vector.md. Briefly, individual mosquitoes were paired‐end sequenced at 30 X coverage and sequencing adapters trimmed before alignment. The paired‐end reads were aligned to the *An gambiae* reference genome (AgamP4) using BWA v.0.7.15. Insertion or deletion realignment was performed using *GATK v.3.7.0 RealignerTargetCreator* and *IndelRealigner* modules. Variants (SNPs) were called using *GATK v.3.7.0 UnifiedGenotyper*, while copy number variants (CNVs) were called based on Gaussian hidden Markov models on normalised call windowed covered data (Lucas et al. 2019).

Samples with coverage below 10× and specimens with evidence of contamination were excluded. A total of 1013 mosquito whole genomes passed quality control. Due to limited samples from the 6, 12 and 18‐month collection time points (PBO arm: *N* = 1, 14 and 1, respectively, while non PBO arm: *N* = 50, 154 and 126), analyses focused on comparisons between baseline (PBO arm: *N* = 185 and non PBO arm: *N* = 207) and 25‐month post‐intervention populations (PBO arm: *N* = 53 and non PBO arm: *N* = 222) (Table S1b).

## 3 Data Analysis

### 3.1 Population Structure

To evaluate the genomic impact of LLIN interventions on the population, we structured the analysis to assess changes in population structure, size and adaptive genomic responses while explicitly addressing the four hypotheses. Population structure was assessed using principal component analysis (PCA) based on 100,000 randomly selected SNPs for each chromosome arm and genome‐wide windowed F_ST_ analysis to assess the degree of genetic differentiation within and between Eastern and Western Uganda populations.

### 3.2 Estimating Effective Population Size

Effective population size (N_e_) was estimated using linkage disequilibrium (LD) analysis (Nei et al. 1975). LD values (r^2^) were calculated for 100,000 randomly sampled SNP pairs between chromosomes 2 and 3 (to exclude physically linked pairs) after excluding singletons, non‐segregating loci and sites with missing data. In chromosome 2, we used these genomic regions (2R:1–17,990,000, 2R:32010000–61,545,105, 2 L:0–19,990,000 and 2 L:43010000–49,364,325), which excluded inverted 2Rb (2R:19023925–26,758,676), 2Rc‐d overlap (2R:26750000–31,473,100), 2La(2 L:20524057–42,165,532) and flanking regions (~0.5–1 Mb).

N_e_ was calculated assuming a recombination rate of 0.5 (because SNP pairs were on separate chromosomes). Confidence intervals were calculated by 1000 iterations of sub‐sampling 90% of the samples and recalculating N_e_. To address H_1_, LD and N_e_ of all pre‐intervention samples were compared with values from size‐matched post‐intervention samples through random subsampling to account for LD's sample size dependency and difference tested using the Mann Whitney U test. For H_2_, populations exposed to PBO‐treated and standard nets were analysed separately, with sample sizes equalised as with H_1_. A second method we used to assess changes in the population size was calculating temporal changes in nucleotide diversity (π), an indicator of recent demographic shifts, using the *malariagen‐data v.13.5* python package (Miles et al. 2024).

### 3.3 Simulating Sample Size Needed to Detect Population Crash in *Anopheles*

To determine the power to detect population crashes at different starting population sizes, we used msprime 1.3.4 (Kelleher et al. 2016) to simulate starting effective population sizes (N_e_) of 100, 1000, 10,000 and 100,000, either remaining at that size or crashing by 10‐fold and remaining at the new population size (10, 100, 1000 and 10,000 respectively) for 25 generations, which is equivalent to the 25 months gap between baseline and round 5 sampling periods we used in our study. In each case, we simulated genomes of contemporary mosquitoes, modelled as two chromosomes of 50 M bp each, with a mutation rate of 10^−8^ and a recombination rate of 10^−8^ per position within each chromosome and 0.5 between chromosomes.

We performed these simulations using both Wright‐Fisher and coalescent models. For coalescent models, we simulated the genomes of 500 individual mosquitoes at each population size. Wright‐Fisher models are more computationally intensive, thus we only simulated the genomes of 250 mosquitoes, and only for starting N_e_ of 100, 1000, 10,000 (a population size of 100,000 being unfeasible to simulate). Population sizes larger than 100,000 were unfeasible to simulate even with the coalescent model. *An. gambiae* population sizes are larger than this (The Anopheles gambiae 1000 Genomes Consortium 2017), and therefore we can only extrapolate to the power of analyses with true *An. gambiae* populations. In each case, we performed 100 iterations of the simulations, except when simulating a population size of 100,000, where we performed 50 iterations to reduce computation. Using the genomes generated by these simulations, we calculated r^2^, and N_e_, using pairs of SNPs between chromosomes, applying the same methods as for the main analysis. From the simulated genomes, we used either all samples, or subsets of samples, to explore the effectiveness of the analysis at the range of sample sizes.

### 3.4 Genome‐Wide Analysis of Change in Allele Frequencies

To test H_3_ and H_4_, we conducted genome‐wide scans comparing baseline and post‐intervention allele frequencies, restricting our analysis to high‐confidence SNPs with good statistical power (biallelic SNPs with a minor allele frequency (MAF) > 5% and no missing data), and passing Ag1000G's site filters (https://malariagen.github.io/vector‐data/ag3/methods.html#site‐filtering). Generalised linear mixed models (GLMMs) were used to test the association between allele frequencies and intervention, including intervention phase (baseline vs. 2 years post‐intervention), geographic location (East vs. West) and net type (PBO nets vs. pyrethroid only nets) as fixed effects and health sub‐districts as a random effect (H_3_) (Brooks et al. 2017). Modelling was performed using the *glmmTMB v.1.1.6* package (Brooks et al. 2017) in *R v.4.4.0* (R Core Team 2019) and *P*‐values for intervention phase were calculated using the drop1 function, applying a Chi square test. Separate models for PBO and pyrethroid nets were fitted to test H_4_, and a Benjamini–Hochberg false discovery rate (FDR) correction was applied (Strimmer 2008). Changes in frequency in SNPs with FDR‐adjusted *p*‐values < 0.05 were considered significantly associated with intervention phase.

### 3.5 Power Simulations for Detecting Adaptive Allele Frequency Shifts

To evaluate our power to detect allele‐frequency differences between rounds, we conducted a simulation‐based power analysis, recreating our datasets with different magnitudes of allele frequency shift. In each simulation, we assumed that 10 SNP loci showed allele frequency changes, with baseline wild‐type allele frequencies sampled from a uniform distribution, and post‐intervention (round 5) frequencies reduced by a factor *e*. Allele counts were generated according to the empirical sample sizes in each round (185 females in baseline; 53 females in post‐intervention in the PBO cohorts, which had the smallest sample sizes), maintaining location and health sub‐district assignments. For each simulated SNP, genotype data were analysed using the binomial GLMM, matching the H_4_ model to obtain the *p*‐value for the round effect. For the remaining 9,898,571 SNP loci (matching the total number of loci used in our analysis minus 10), we drew *P*‐values from a random uniform distribution, as expected from the null hypothesis of no association. We applied FDR correction across all *P* values. This simulation was repeated 500 times per value of e, and power was defined as the proportion of selected SNPs detected at FDR < 0.05, reporting the mean and 95% confidence intervals. We simulated values of e equal to 1, 1.1, 1.2, 1.3, 1.4 and 1.5 to identify the effect size that we were able to detect.

### 3.6 SNP‐Cis Regulatory Element Mapping and Enrichment Analysis

To determine whether SNPs exhibiting significant frequency shifts during the trial were enriched within cis‐regulatory elements (CREs), we mapped all GWAS variants to experimentally validated regulatory regions using ATAC‐seq data from *Anopheles gambiae* (Ruiz et al. 2021). CRE coordinates and annotations were obtained from the ATAC‐seq peak catalogue reported in the supplemental dataset of Ruiz et al. (2021) (Table S4), which provides transcriptionally active regulatory regions defined by peak summits, gene assignments and consensus regulatory classifications generated by HOMER and ChIPseeker. Peaks were retained together with their genomic coordinates, regulatory annotation (“Annotation final”) and gene associations (“Annotated Gene ID final”).

All SNPs from the allele‐frequency change GWAS (comparing pre‐ and post‐LLIN distributions) were first converted to the BED format, and base‐pair overlaps with ATAC‐seq peaks were identified using *BEDTools v.2.30.0 intersect* (Quinlan and Hall 2010). Where individual SNPs overlapped multiple peaks, we collapsed these to a single CRE assignment using a fixed functional hierarchy (Promoter → 5′UTR → Exonic → Intronic → Downstream bins (form nearest to translation start site (TSS) or ATG) → Distal Intergenic), breaking ties using proximity to the gene TSS/ATG. SNPs with no overlap were classified as “non‐THS (Transposase hypersensitive sites)”. To quantify regulatory enrichment, two complementary nonparametric approaches were applied. **(i) Rank‐based enrichment:** All SNPs were ranked by the absolute GWAS score (−log10 *p*). For each CRE category, we computed the Mann–Whitney U‐derived AUC statistic, interpreted as the probability that a CRE‐assigned SNP has a higher score than a randomly chosen non‐CRE SNP.

**(ii) Permutation‐based enrichment:** To evaluate whether high‐scoring SNPs were enriched within CREs, we permuted CRE annotations across all SNPs (1000 iterations), preserving the empirical frequency of each annotation and recomputing the number of CRE‐linked SNPs among the top SNPs (*p* < 0.001). Empirical *p*‐values were derived from the null distribution of permuted counts. Finally, genes linked to top‐scoring CRE SNPs were extracted using peak‐to‐gene assignments provided in the ATAC‐seq dataset.

### 3.7 Window‐Based Genome Wide Analysis of Change in Haplotype Frequency

A window‐based approach using H12 statistics was applied as a more powerful but less precise method to identify genomic regions undergoing selective sweeps and adaptive responses. H12 statistics were calculated for phased haplotypes in 1000‐SNP sliding windows. ΔH12, defined as the difference in H12 values between post‐ and pre‐intervention populations, was used to detect regions undergoing changes in haplotype frequencies. Positive ΔH12 values indicated reduced haplotype diversity post‐intervention, suggesting increased selective pressure after the intervention, while negative values reflected increased haplotype diversity. To identify putative selective sweep regions, we first defined peaks as windows with ΔH12 values exceeding three times the difference between the median and the 98th percentile of the genome‐wide ΔH12 distribution (Lucas et al. 2023). To assess the statistical significance of these peaks, we performed 1000 random permutations and recalculated ΔH12 for each permuted dataset. At each genomic window, empirical *p*‐values were obtained by comparing the observed ΔH12 to the null distribution of permuted values.

To identify and describe the haplotypes underlying significant signals of change over the intervention, we used hierarchical clustering of phased haplotypes using pairwise genetic distances (dxy). In each of the peaks where ∆H12 changed significantly, we carried out the hierarchical clustering using SNPs within the genomic region encompassing 5% decay on both sides from the centre of the H12 peak (Figure S1). The centre of the peak was determined by fitting an exponential model on the calculated H12 to find the peak of the curve (https://malariagen.github.io/agam‐selection‐atlas/0.1‐alpha3/index.html). Clusters of haplotypes forming a selective sweep were identified by cutting the tree using a threshold of dxy = 0.001. Each cluster was then considered a haplotype allele. Associations between haplotype allele frequencies and intervention phases were assessed with GLMMs to address hypotheses H3 and H4. We present the nominal *p*‐value from each glmm analyses and fdr corrected *p* value (corrected based on all *p*‐values from all loci).

We tested associations between these haplotype clusters and CNV calls at key metabolic resistance loci *Cyp6aa*‐*Cyp6p* and *Cyp9k1*. Because the CNV data are not phased, we identified tagging SNPs for the different CNV alleles at these loci through Spearman rank correlation of SNP calls and CNV calls at the sample level. For each CNV allele, the SNP with the highest correlation coefficient was used as a haplotype‐level proxy for the CNVs.

### 3.8 Data and Code Availability

All samples were obtained from the LLINEUP trial, which was approved by the Ugandan National Council for Science and Technology (UNCST Ref HS 2176) and the Liverpool School of Tropical Medicine (Ref 16–072). The epidemiological data from the LLINEUP trial can be accessed on ClinEpiDB (LLINEUP Cluster Randomized Trial). The variant data (SNPs and CNVs) used in this study are available through the MalariaGEN *Anopheles gambiae* 1000 Genomes Project (Ag1000G) and can be accessed using the malariagen‐data Python package (https://malariagen.github.io/malariagen‐data‐python/v13.0.0/) or downloaded at https://malariagen.github.io/vector‐data/ag3/download.html. The sample sets used are [“1288‐VO‐UG‐DONNELLY‐VMF00168”, “1288‐VO‐UG‐DONNELLY‐VMF00219”]. All code used for the analyses in this study is available in our public GitHub repository at https://github.com/Harunnjoroge/llineup_publication. This repository contains scripts for data processing, population genetic analyses and figure generation, ensuring full reproducibility of our results.

## 4 Results

### 4.1 Pre‐Intervention Genetic Structure of *Anopheles gambiae* in Uganda

Principal component analysis (PCA) revealed no clear genetic differentiation between Eastern and Western Uganda populations (Figure S2A). No structure was observed for any chromosome except for that associated with the 2Rb and 2La chromosomal inversion regions. Inclusion of these regions revealed genetic structure by inversion genotypes, but not between East and West populations, confirming a high degree of genetic homogeneity across regions.

Nevertheless, weak isolation by distance may still exist, which is not strong enough to be detected by PCA. To test this, we calculated windowed F_ST_ between the geographical regions. We found localised significant differentiation in all the chromosomes, particularly around genes linked to pyrethroid resistance and the 2Rb and 2La inversions, suggesting a more rapid evolution within these loci than gene flow between the regions in Uganda (Figure S2B and Table S2). These results suggest that, while gene flow is extensive, regional differences exist at loci of recent selection. To account for this differentiation, location was incorporated as a fixed effect in subsequent generalised linear mixed models (GLMMs).

## 5 H1 and H2, Genetic Analysis of Population Size Change After Bed Net Intervention

While the intervention led to a significant decline in indoor *An. gambiae* catch numbers in both arms of the LLINEUP trial in the first year and possible continued decline in the PBO arm (Table S3), this decline was not detected in the genetic analysis of effective population size (Maiteki‐Sebuguzi et al. 2023). Linkage disequilibrium (LD) is expected to increase as the effective population size declines due to more recent shared ancestry (Hollenbeck et al. 2016) but we found no significant differences in LD or in the LD‐based N_e_ between baseline and post‐intervention periods both with and without stratification by net type (Table S4). *An. gambiae* typically exhibits extremely low LD and very large N_e_ in the order of tens of millions (Khatri et al. 2019; The Anopheles gambiae 1000 Genomes Consortium 2017). In this study, N_e_ estimates derived from LD were highly variable, with upper 95% confidence intervals extending to infinity and lower confidence intervals below 5000, reflecting the lack of precision in Ne estimates of very large populations. Such high N_e_ estimates were found both before and after intervention, indicating that despite the intervention, populations remain too large for accurate estimation of changes in N_e_ through the LD‐based method. Similarly, nucleotide diversity showed no significant differences between baseline and post‐intervention periods (Table S4).

To determine the power of our genomic analysis to detect changes in N_e_, we conducted simulations of 10‐fold population crashes with different starting population sizes. For small populations (starting N_e_ = 100), median N_e_ estimates clearly distinguished crash from no‐crash scenarios, providing > 80% power with as few as 75 mosquitoes. At N_e_ = 1000, separation between crash and non‐crash estimates remained robust, with > 80% power to detect change with 100 mosquitoes (Figure S3).

For larger populations (N_e_ = 10,000), N_e_ estimates became increasingly variable, and the 97.5% centile was frequently infinite at small sample sizes (Figure S3a). Nevertheless, the median N_e_ values for crash and non‐crash scenarios remained distinguishable with 80% power for 150–200 samples. At N_e_ = 100,000, both scenarios frequently produced infinite upper confidence limits even with 500 samples. Detection power remained low for N_e_ = 100,000 unless ≥ 400–500 genomes were sampled (Figure S3b). Together, these results indicate that LD‐based N_e_ estimation is sensitive to population decline in small to moderately large populations, but even a 10‐fold change in population size is unlikely to be detected in the large populations found in *An. gambiae*.

## 6 H3 and H4, Genome‐Wide Association Analysis Reveals Net Type‐ and Population‐Specific Genetic Changes

Genome‐wide SNP analysis identified 21 SNPs that exhibited significant frequency changes over the course of the intervention across the study sites (H3). Among these, a nonsynonymous SNP (2 L:10334049, A>C, V390G) was detected in *AGAP005127*, which encodes RNA‐binding protein 15. Seven SNPs were intronic, located within *CYP4G16* (X:22941281 and X:22941291), *AGAP029982* (3R:34361036), *C‐type lectin* (X:18216512, X:18216520, X:18216526) and *Fatty acyl‐CoA reductase* (X:23560372). The remaining SNPs were intergenic (Figure S4).

The intergenic SNPs were in the following regions on the genome; on chromosome 2R, five SNPs (2R:59036522 to 2R:59036642) were located 14 kb upstream of *Juvenile hormone diol kinase*, while two SNPs (2R:60709765 and 2R:60709812) were positioned 3.5 kb downstream of *AGAP004665* (a *CYP306A1*‐like gene). Another SNP (2R:61318911) was identified 53 kb upstream of *AGAP004675*, a putative *muscarinic acetylcholine receptor 2*. Additional intergenic SNPs included two loci on chromosome 3R (3R:51526360 and 3R:51526384), located 3.7 kb upstream of *AGAP010243*, one SNP on 3 L (3 L:745991), positioned 24 kb upstream of *AGAP010324* and another on 3 L (3 L:4240178), found ~4.5 kb upstream of *AGAP010481*. On the X chromosome, SNP X:20244647 was located 6.5 kb upstream of *AGAP013064*.

Out of all the significant intergenic SNPs, only 3 L:4240178 overlapped a regulatory element. This SNP fell within a distal intergenic ATAC‐defined region located ~4.5 kb upstream of *AGAP010481*, a solute carrier family 6 (SLC6) transporter gene. Across the genome, SNPs located within cis‐regulatory elements showed no rank‐based enrichment relative to non‐CRE SNPs (AUC range 0.493–0.509, where 0.5 refers to no difference and over or below 0.5 indicates direction of enrichment). Permutation testing of the top GWAS hits (SNPs with raw *p* < 0.001) permuted 1000 times; preserving the empirical frequency of each CRE annotation and recomputing the number of CRE‐linked SNPs among the top SNPs (*p* < 0.001) did not detect significant over‐representation of any CRE category after controlling for the background frequency of annotations (empirical *p* > 0.22 for all functional classes). We note that many of these loci are within or close to centromeres. Centromeric loci have low recombination, extended LD and potential artefacts due to repetitive structure in this region; thus, these hits should be interpreted cautiously.

In H4, we hypothesised that net‐specific changes in SNP frequencies would be observed; however, no significant SNP frequency changes were detected when the trial arms were analysed separately. Splitting our samples by intervention arm (see Table S1b), we will have reduced our power to detect shifts in SNP frequency. Our smallest sample size was in the PBO cohort (*n* = 185 baseline; *n* = 53 post‐intervention), and our power simulations revealed limited power to detect small changes in allele frequency (Figure S5). However, this sample size provided 80% power to detect a wild‐type allele frequency fold‐reduction of 1.4 (i.e., a 29% reduction in wild‐type allele frequency from baseline to post‐intervention). These results indicate that, although small allele‐frequency shifts may remain undetected, the available sample sizes are sufficient to reliably detect moderate to strong adaptive changes.

**FIGURE 1:**
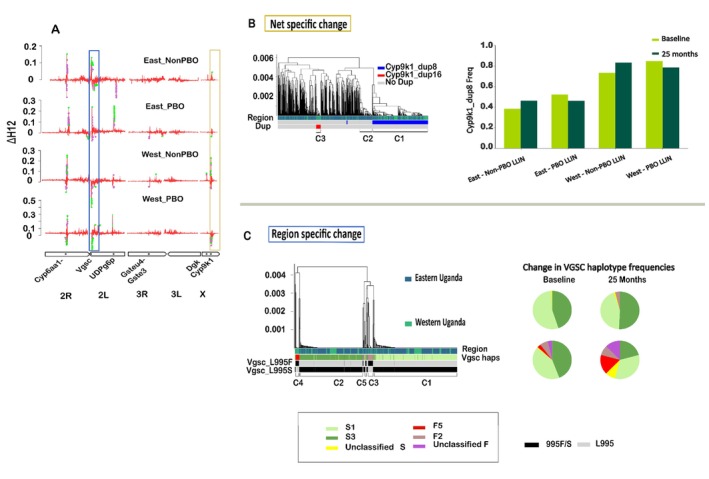
Targeted genomic responses to bed net interventions identified by genome‐wide haplotype homozygosity (ΔH12) in Anopheles gambiae during the LLINEUP trial in Uganda. (A) Genome‐wide ΔH12 estimates (H12_end_–H12_baseline_) across four experimental cohorts from the LLINUP trial, plotted by the chromosome arm. Positive ΔH12 values reflect reduced haplotype diversity post‐intervention, suggesting directional selection. Coloured dots mark windows surpassing the permutation‐based significance threshold (green: Significant; purple: Marginal). Labelled boxes denote regions of interest (blue: Region specific change; Khaki: Bed net specific change). (B) Dendrogram of haplotypes at the Cyp9k1 locus on chromosome X, highlighting net‐specific selection. Distinct haplotypes associated with the Cyp9k1 duplication (dup8, dup16) cluster with a lineage showing decreased frequency in PBO‐treated cohorts and vice versa in non PBO‐treated cohorts (see ΔH12 peaks in panel A). The bar plot (top right) tracks Cyp9k1 duplication frequency changes by region and treatment. (C) Dendrogram of the Vgsc locus on chromosome 2 L, showing the region‐specific haplotype structure. Haplotype clusters correspond to known resistance alleles (Vgsc‐L995F and Vgsc‐L995S) and their haplotype backgrounds. Pie charts illustrate temporal shifts in Vgsc haplotype composition across regions and treatments, corresponding to ΔH12 dynamics in panel A.

To complement single‐SNP analyses, we utilised window‐based methods (∆H12) to detect broader signals of selection. We subtracted the H12 values before the intervention from those after 25‐month post‐bed net distribution to obtain the ∆H12. The genomic regions that changed significantly were determined from random permutations (1000 times) and the recalculation of ΔH12 for each permuted dataset. Empirical *p*‐values were obtained by comparing the observed ΔH12 to the null distribution of permuted values. This revealed 5 key genomic regions where significant changes in haplotype frequencies occurred during the intervention (Figure 1 and Figure S1): 2R:28463444–28,499,726, 2 L:2791320–2,893,275, 2 L:34081017–34,101,131, X:9179019–9,185,374 and X:15216225–15,271,654. A net‐specific change was clearly demonstrated in the X:15216225–15,271,654 region, which encompasses *Cyp9k1*, where ∆H12 showed a significant positive signal in both cohorts (Eastern and Western Uganda) that received standard bed nets, but the reverse was observed in both PBO bed net cohorts (Figure 1). Haplotype clustering analysis linked the observed net‐specific change to the main sweep haplotype allele in the X:15216225–15,271,654 genomic region, which significantly increased in frequency (FDR‐corrected *p* = 0.04) in cohorts that received standard bed nets (Table S5). This haplotype was associated with an increase in the *Cyp9k1* copy number caused by the CNV allele *Cyp9k1*_Dup8 (Figure 1). Accordingly, *Cyp9k1_*Dup8 increased in frequency in both cohorts that received standard bed nets (East 43% to 51% and West 84% to 93%) but decreased (East 56% to 54% and West 94% to 79%) in both PBO cohorts.

**FIGURE 2:**
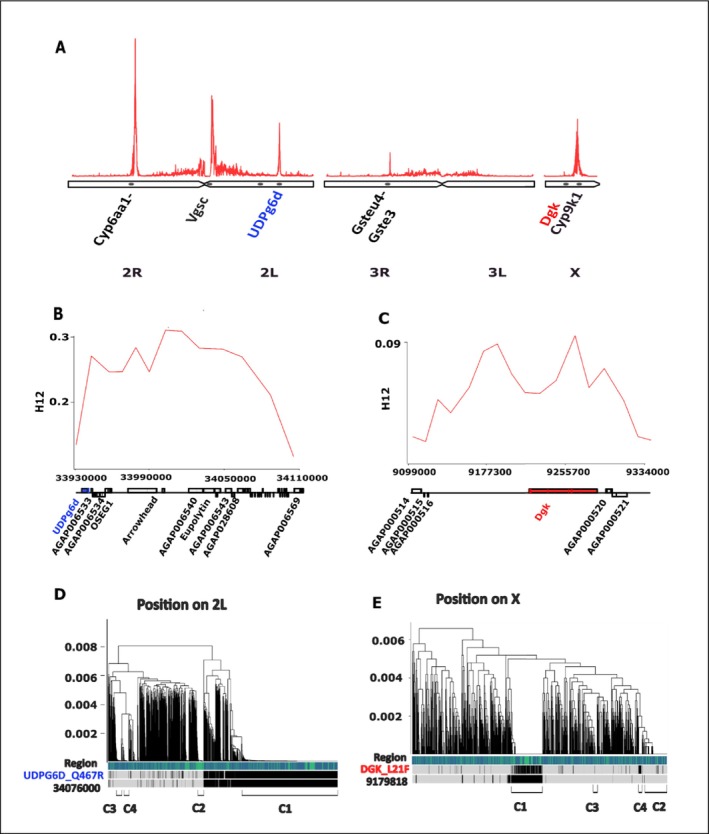
Novel selective sweeps impacted by bed net interventions reveal putative drivers of resistance evolution in ***Anopheles gambiae*** . (A) Genome‐wide H12 scan of Ugandan An. gambiae populations identifies regions under recent selection. In addition to known insecticide resistance loci, a prominent novel sweep on chromosome 2 L (~34 Mb) includes the metabolic gene UDP‐glucose 6‐dehydrogenase (UDPg6p), and a sweep on the X chromosome (~9.2 Mb) spans the diacylglycerol kinase (Dgk) gene. (B–C) Local H12 profiles for the 2 L (B) and X (C) regions show elevated haplotype homozygosity across windows surrounding the candidate genes. (D–E) Dendrograms of haplotypes in the same 2 L (D) and X (E) sweep regions highlight the dominant haplotype clusters associated with the selective sweeps. Candidate genes and putative tagging SNPs are annotated below each panel. Figures B–C and D–E have different scales on the Y axes.

Similarly, the X:9179019–9,185,374 locus also showed positive ∆H12 in standard bed net cohorts, which was significant in Eastern Uganda, but no significant change in the PBO net cohorts from both locations in Uganda (Figure 1 & Table S5). The main haplotype in this region significantly increased in frequency in the overall population (H3: FDR‐corrected *p* = 0.003), but this change was driven by the standard bed net cohorts (H4: FDR‐corrected *p* = 0.0005), while in PBO bed net cohorts, the change was not significant (Table S5). This genomic region lies within the intergenic region between *AGAP000516* (Enhancer of rudimentary protein) and *AGAP000519* (ATP‐dependent Diacylglycerol Kinase (*Dgk*)) (Figure 2). Neither of the genes has copy number variants, but *Dgk* has a missense mutation (L21F), which is associated with the swept haplotype (Figure 2). The sweep is also associated with other non‐coding SNPs from the genomic region (Table S6).

A change in frequency in a swept haplotype within the 2La chromosomal inversion on chromosome 2 L (2 L:34081017–34,101,131) also followed a net‐specific trend, but opposite to that observed in the two regions on the X chromosome (Figure 1). A significant increase in the main haplotype allele (FDR‐adjusted *p* = 0.003) occurred in cohorts that received PBO bed nets, whereas no significant change was detected in the standard bed net cohorts. The main haplotype is not linked to copy number variations, which are absent in the genes within the region; however, a missense SNP in 2 L:33945793 (AGAP006532_Q467R) in *UDP glucose 6 dehydrogenase* (Figure 2) and other SNPs were associated with the haplotype (Table S6).

The 2R:28463444–28,499,726 and 2 L:2791320–2,893,275 swept haplotype changes were not net‐specific, aligning with the H3 hypothesis (Figure 1). In both bed net cohorts, the swept haplotypes in the 2R:28463444–28,499,726 genomic region, where the *Cyp6aa1‐Cyp6p2* gene cluster is located, increased in frequency (Figure 1). Recently, a major haplotype linked to a duplication in the *Cyp6aa1* gene (Figure S6), a SNP (I236M) in the *Cyp6p4* gene, and an insertion of a transposable element were identified in the same genomic region. The haplotype is highly associated with pyrethroid resistance in East and Central Africa (Njoroge et al. 2022). Despite having significant positive ∆H12 (Figure 1), the main haplotype did not significantly increase in frequency in both bed net cohorts during the intervention, but the second most common haplotype, which lacks duplication of the *Cyp6aa1* gene (Figure S6), decreased significantly (FDR adjusted *p* = 0.02) in frequency (Table S5) implying it was less advantageous in the presence of the interventions.

In the 2 L:2791320–2,893,275 region around the *Vgsc* gene, the main haplotypes (S1 and S3 (Clarkson et al. 2021)), which are associated with the *Vgsc‐*995S mutation (Figure 1), increased in frequency in both PBO and standard bed net cohorts in Eastern Uganda, while the opposite trend occurred in Western Uganda, where a minor haplotype (F5) linked to *Vgsc*‐995F (Figure 1) increased in frequency as the main (*Vgsc‐*995S) haplotypes declined (Tables S5 and S7). This *Vgsc*‐995F haplotype was confined to Western Uganda (Figure 1) where it increased in frequency from 2% to 8.3% in cohorts that received standard bed nets, while in PBO cohorts, it increased from 2.7% to 14.3%.

## 7 Discussion

This study provides a genomic insight into the adaptive responses of *An. gambiae* populations to large‐scale bed net interventions, highlighting the selective pressures exerted by insecticide‐based vector control. Using genome‐wide SNP analyses, haplotype clustering and diversity metrics, we provide evidence of population‐specific and intervention‐specific genetic changes, particularly at loci associated with pyrethroid resistance.

### 7.1 Genetic Diversity and Population Structure

Contrary to our initial expectations based on the entomological outcomes of the LLINEUP trial (Staedke et al. 2020; Maiteki‐Sebuguzi et al. 2023), the genetic indicators of population size (nucleotide diversity (π), linkage disequilibrium (LD) or LD‐based effective population size (N_e_)) did not show evidence of a decline in population size following the intervention. When we compared the baseline population and 25 months post bed net distribution, the nucleotide diversity and LD remained stable in both standard and pyrethroid‐PBO net cohorts. The LD‐based N_e_ estimates were unreliable as the confidence interval boundaries included infinity in both time periods; hence, a change in population size could not be confirmed. This stands in contrast to the substantial declines in indoor Anopheles densities observed across all study arms, particularly those receiving PBO nets.

The LD method is most accurate when the population size is small and becomes increasingly inaccurate in large populations, characteristic of *An. gambiae* (Leffler et al. 2012; The Anopheles gambiae 1000 Genomes Consortium 2017). In such cases, the method often fails to provide meaningful estimates, frequently reporting infinite N_e_ values. Similar limitations have been observed in other studies estimating N_e_ in *Aedes aegypti* , *Culex quinquefasciatus* and the Miami blue butterfly, where LD‐based estimates often yielded unrealistic values compared to temporal and coalescent‐based approaches (Saarinen et al. 2010; Saarman et al. 2017; Huang et al. 2023).

Alternative approaches that leverage adaptation dynamics, such as estimating N_e_ from the number of independent mutational origins contributing to a soft selective sweep, can yield finite N_e_ estimates in *An. gambiae*. Applying this approach to the insecticide‐resistance locus *Vgsc* in *An. gambiae*, Khatri et al. 2019, estimated very large N_e_ on the order of 10^7^ to 10^9^. Importantly, this soft‐sweep estimator is inherently locus‐ and selection‐trajectory specific. It reflects the effective population size relevant to the rise of a particular adaptive allele, given assumptions about mutation rates and allele‐frequency dynamics, rather than genome‐wide drift across neutral loci. As the frequency of the VGSC L995S/F mutations in Uganda is very high and nearly fixed in most regions (Lynd et al. 2019), we opted to use the LD‐based *N*e, which is broadly applicable without requiring an identifiable selective event and is commonly used as a single‐sample estimator across many loci presumed to be selectively neutral and unlinked (Waples and England 2011). Considering the indoor densities of Anopheles significantly reduced during the trial, we expected the population would have been reduced enough, especially in pyrethroids‐PBO cohorts, to be reliably detected by LD‐based N_e_ estimation.

Our simulation analyses showed that for populations with starting Ne = 100,000, still considerably smaller than contemporary estimates for *An. gambiae* (The Anopheles gambiae 1000 Genomes Consortium 2017), even samples of 500 genomes detected crashes in only a minority of simulations, and both 10× crash and no‐crash scenarios routinely produced unbounded N_e_ estimates. Our results indicate that the size of *An. gambiae* populations is such that an intervention sufficient to reduce both mosquito catch numbers and malaria transmission can have an indiscernible impact on the population's genetic diversity, even if the change in population size is 10‐fold. This may explain the recovery of catch numbers within two years of bed net deployment.

The steady population despite the deployment of control tools can result from high gene flow, as evidenced by PCA and differentiation analyses in our study, suggesting that minimal barriers to inter‐regional migration may have allowed for the replenishment of localised diversity losses. Alternatively, the decline in trapped mosquitoes resting indoors as reported in the LLINEUP trial (Maiteki‐Sebuguzi et al. 2023) may also result from behavioural avoidance driven by interventions, including shifts in feeding or resting preferences, which have been observed in other field studies (Prussing et al. 2018; Degefa et al. 2021; Machani et al. 2022; Omondi et al. 2023).

### 7.2 Locus‐Specific Selection Pressures

Although genetic diversity and effective population size did not show detectable changes, our results show that the population was nonetheless impacted by interventions, with selection acting on specific loci or haplotypes. Significant changes in allele frequencies at resistance‐associated loci highlight the intense selective pressures imposed by bed nets. Regions encompassing genes known to be associated with resistance, such as the pyrethroid target site *Vgsc* (Grigoraki et al. 2021), and metabolic resistance gene regions *Cyp6aa1‐Cyp6p2* and *Cyp9k1* (Vontas et al. 2018; Njoroge et al. 2022) exhibited patterns consistent with positive selection, aligning with their established roles in target‐site and metabolic resistance mechanisms.

For instance, the increased frequency of the main haplotype in the *Cyp6aa1‐Cyp6p2* region, which co‐occurs with *Cyp6aa1* duplication and *Cyp6p4*‐*236 M* mutations, underscores its adaptive significance. This haplotype, previously identified in East and Central Africa, confers enhanced metabolic detoxification capacity, facilitating survival in the presence of pyrethroids (Njoroge et al. 2022). Similarly, the dynamics at the *Vgsc* locus reveal the persistence of knockdown resistance alleles. Though our sample size was modest (Table S1), the observed shifts in *Vgsc* haplotypes corroborate a model showing the flow of the *Vgsc‐995F* from the west into Western Uganda (Lynd et al. 2024). We used the same tagging SNPs and haplotype allocation method as Lucas et al. (2019) to identify the actual knockdown resistance haplotypes that were changing in frequency during the intervention. These origins include S1 to S5, which are the different haplotype backgrounds of the *Vgsc*‐995S mutation, and F1 to F5, the haplotype backgrounds of *Vgsc*‐995F. In East Africa, the main haplotypes are S1 and S3. The haplotype background analysis revealed the F5 haplotype, which was originally identified in Cameroon, Gabon and Democratic Republic of Congo (Lucas et al. 2019; Clarkson et al. 2021), increased in frequency during the intervention in Western Uganda, while S1 and S3 haplotypes decreased in frequency (Figure 1). The replacement of *Vgsc‐995S* haplotype backgrounds with *Vgsc‐995F* haplotype reflects the adaptive advantage of the latter (Lynd et al. 2010), consistent with its near fixation of *Vgsc‐995F* in West African populations highly resistant to pyrethroids (Kouamé et al. 2023).

### 7.3 Net‐Specific Selective Pressures

We observed significant net‐specific changes in the frequencies of major alleles across three genomic regions: *2La‐34 Mb* (2 L:34,081,017–34,101,131), *AGAP000516‐Dgk* (X:9,179,019–9,185,374) and *Cyp9k1* (X:15,216,225–15,271,654). Notably, the frequency of the primary haplotype at *2La‐34 Mb* increased significantly in the intervention arm that received PBO + pyrethroid bed nets, suggesting that PBO nets may impose a distinct selective pressure on this locus. Although a selective sweep in the broader 2La region has been reported previously, the selective agent underlying that sweep remains unresolved (The Anopheles gambiae 1000 Genomes Consortium 2017). The rise of this haplotype frequency during the PBO intervention is consistent with the hypothesis that adaptive variants at *2La‐34 Mb* provide alternative resistance mechanisms when cytochrome P450‐mediated detoxification is inhibited by PBO.

The 2La chromosomal inversion is primarily associated with thermal and desiccation tolerance (Gray et al. 2009; Rocca et al. 2009). Because recombination is reduced between alternative karyotypes, 2La can maintain extended haplotypes and co‐adapted alleles in partial linkage disequilibrium, sometimes yielding supergene‐like behaviour in association analyses (White et al. 2007). Consequently, alleles affecting insecticide resistance can segregate with the inversion state rather than as independent loci. For example, resistance to dieldrin (and related compounds such as fipronil) has been reported to associate strongly with 2La karyotype in *An. gambiae* populations from West Africa (Brooke et al. 2000). Subsequent work clarified that this association reflected the distribution of resistance mutations (e.g., 296G/S) in the GABA‐gated chloride channel gene (*Rdl*), which lies within the 2La inversion (Adeogun et al. 2019; Grau‐Bové et al. 2020). However, the *GABA* gene (2 L:25,363,652–25,434,556) lies outside the swept region identified here, and the 296G/S mutations are absent in our population, making it unlikely that *Rdl* contributed directly to the signal of positive selection we detect at 34 Mb.

There is also evidence that the inversion state may influence insecticide exposure via cuticular traits. Mosquitoes carrying the standard arrangement (2 L + a) have been reported to exhibit thicker cuticles, which may reduce water loss (Reidenbach et al. 2014), and could also impede insecticide uptake (Balabanidou et al. 2016). This observation aligns with findings from Kenya, where the frequency of the standard chromosomal form increased from 0% to 76% between 1994 and 2011, coinciding with the rise in pyrethroid bed‐net coverage from 0% to over 90%. This change occurred independent of climatic factors (Matoke‐Muhia et al. 2016). Similarly, in our Ugandan population, the swept haplotype was found exclusively in mosquitoes carrying the standard 2La chromosome (Figure S7). This tight linkage makes it difficult to disentangle whether the increase reflects selection on the swept haplotype itself or a broader shift in the frequency of the standard 2 L + a arrangement. Among the genes within the swept region, none are directly linked to cuticle metabolism, making it unlikely that the sweep is associated with cuticle thickening. However, *UDP‐glucose 6‐dehydrogenase*, a gene involved in detoxification, is located within this region. This gene encodes an enzyme that catalyses the oxidation of UDP‐glucose to UDP‐glucuronic acid, which is subsequently conjugated by UDP‐glucosyltransferases with endogenous or exogenous compounds, facilitating their deactivation or excretion (Real et al. 1991; Hung et al. 2007) Recent functional work suggests that this broader conjugation pathway contributes to pyrethroid detoxification in *An. gambiae* and *An. funestus* (Logan et al. 2024). Thus, this pathway may be an important alternative when cytochrome P450 enzymes are inhibited by PBO, providing an explanation for the increased frequency of this haplotype.

In contrast, standard pyrethroid‐only nets maintained selective pressures favouring metabolic resistance, as evidenced by changes in the *Cyp9k1* region. *Cyp9k1*, a cytochrome P450 gene, is a key pyrethroid metabolizer, explaining the increased frequency of this haplotype during the intervention (Vontas et al. 2018). This gene is duplicated in Uganda, where the main CNV allele is *Cyp9k1*_Dup8 with a secondary minor allele, *Cyp9k1_*Dup16. We found that *Cyp9k1*_Dup8 is fully linked to the main haplotype that was increasing in frequency during the bed net intervention (Figure 1). The elevated copy number in *Cyp9k1* likely results in additional enzyme copies, enhancing the overall capacity for pyrethroid metabolism and thus increasing resistance. However, in the presence of PBO, which inhibits cytochrome P450 enzymes (Jones 1998), additional copies of *Cyp9k1* may offer no advantage. This could explain the observed negative selection of the haplotype in populations exposed to PBO + pyrethroid bed nets (Figure 1). Our findings suggest that PBO may disable *Cyp9k1* detoxification of pyrethroids sufficiently that selection on this gene is removed or reversed, rather than enhanced to overcome the inhibition. However, alternative mechanisms, such as the proposed involvement of UDP‐glucosyltransferases, may be recruited to compensate for this loss.

A novel swept locus X:9,179,019–9,185,374 on the X chromosome also exhibited a significant increase in frequency over the intervention in the pyrethroid‐only cohort. Unlike the *Cyp9k1* region, no opposite change was detected in the PBO + pyrethroid cohorts. This locus is flanked by several genes: *Agap000513* (*dipeptidase E), Agap000515, Agap000516* (*Enhancer of rudimentary protein*) and *Agap000519* (*diacylglycerol kinase, Dgk*), none of which have been definitively shown to play a direct role in adaptation to pyrethroids. However, there are reasons to believe that *Dgk* may play a role in resistance. It is involved in modulating levels of secondary lipid messengers, such as diacylglycerol (DAG) and phosphatidic acid (PA), which influence key biological processes, including neurotransmission, lipid metabolism, protein kinase C activity, vesicle trafficking and the regulation of membrane protein function (Topham 2006). The roles of *Dgk* in neurotransmission and vesicle trafficking may indirectly contribute to pyrethroid resistance.

In mammalian synapses, knockdown of the *Dgkθ* gene resulted in slow recovery of synaptic vesicles following stimulation, and rescue of normal recycling kinetics required *Dgk* catalytic activity, directly linking its function to maintaining synaptic transmission during periods of elevated neuronal activity (Goldschmidt et al. 2016). Pyrethroids disrupt nervous system function by modifying voltage‐gated sodium channel gating, driving sustained neuronal hyperexcitability and ultimately conduction failure (Soderlund 2012; Soderlund and Knipple 2003). Under repeated or sublethal exposure as expected in LLIN contact scenarios, variation affecting vesicle recycling capacity could plausibly support rapid recovery of neurotransmission after knockdown contributing to resistance to pyrethroids.

### 7.4 Implications for Resistance Management and Future Directions

Our findings emphasise the interplay between intervention type and mosquito genetic responses. The persistence of resistance‐associated alleles, even with novel nets containing a resistance‐breaking synergist, highlights the difficulty of reversing entrenched adaptations once they are common in a population. At the same time, the identification of loci such as *Dgk* and SNPs in introns or intergenic regions (with no established resistance annotation) points to additional adaptive routes beyond canonical target‐site or metabolic detoxification mechanisms, including changes in neuronal resilience, behaviour or cis‐regulatory control of gene expression.

For example, the swept region harbouring *Dgk* is consistent with selection acting on synaptic membrane trafficking rather than detoxification per se. Diacylglycerol kinases regulate DAG/PA signalling at membranes and have been directly implicated in presynaptic vesicle endocytosis/recycling, where loss of *Dgkθ* slows vesicle retrieval and delays recovery of synaptic vesicle pools following stimulation (Goldschmidt et al. 2016). In the context of pyrethroid exposure where intoxication is dominated by neurophysiological disruption, standing variation that improves vesicle recycling capacity could plausibly facilitate functional recovery after sublethal contact, shifting survival without requiring increased metabolic clearance.

In addition, one SNP (3 L:4,240,178) that changed significantly during the intervention maps to a distal cis‐regulatory element upstream of *AGAP010481*, annotated as an *SLC6* transporter. Members of the SLC6 family include transporters that regulate neurotransmitter homeostasis by mediating reuptake/clearance and transport amino acids/osmolytes that contribute to neuronal and metabolic homeostasis (Pramod et al. 2013). The decline in frequency of this SNP following LLIN distribution may therefore reflect negative selection acting on a haplotype that perturbs solute transport and downstream neuronal physiology. Beyond mosquitoes, solute carrier transporters have also been implicated in xenobiotic tolerance in agricultural insects, suggesting they can contribute to pyrethroid resistance through changes in uptake, distribution or physiological buffering (Jin et al. 2025). This warrants targeted follow‐up to resolve the regulatory effects and phenotypic consequences in *An. gambiae*.

Overall, the persistence of resistance associated markers and signature of selection in novel regions emphasises the need for deployment of diversified, integrated vector control strategies (including IRS with non‐pyrethroids, eave tubes, house improvement and larval source management) in populations with elevated pyrethroid resistance. While this study provides a comprehensive analysis of genetic responses to bed net interventions, certain limitations should be noted. Intermediate changes at 6‐, 12‐ and 18‐months post‐intervention could not be analysed due to limited sample sizes. This is the period when the biggest drops in catch numbers were seen and likely the highest chance of finding a drop in N_e_. Key changes may have been missed due to gene flow from non‐intervention regions or reduced selection pressure as insecticide efficacy waned. Expanding the temporal and spatial scope of sampling is essential for a comprehensive understanding of resistance dynamics.

## 8 Conclusions

Genomic surveillance should remain central to malaria control programs, enabling the early detection of emerging resistance alleles and informing adaptive strategies (Weetman and Donnelly 2015). In regions like Uganda, where *An. gambiae* populations exhibit both high genetic homogeneity and region‐specific selective pressures, targeted interventions accounting for these dynamics could sustain control measures' efficacy. By integrating genomic surveillance with epidemiological and entomological monitoring, we can develop more sustainable, evidence‐based strategies to combat malaria and insecticide resistance. Our findings underscore the advantages of WGS in identifying adaptive responses missed by approaches that use only known resistance markers (Lynd et al. 2024) emphasising the importance of being integrated in control programs. Tracking changes in population structure, diversity and resistance allele frequencies provides early warning signals of control failure, guiding the strategic deployment of resistance management tactics in an era of rising resistance (Neafsey et al. 2021).

## Author Contributions

Harun N. Njoroge, Grant Dorsey, Janet Hemingway, Sarah G. Staedke, Eric R. Lucas and Martin J. Donnelly designed the study; Anastasia Hernandez‐Koutoucheva, Chris Clarkson and Alistair Miles processed the raw whole genome sequences (alignments, variant calling and haplotype phasing) to analysis‐ready data available through the MalariaGen API. Harun N. Njoroge, Lilian Namuli, Sanjay C. Nagi, Eric R. Lucas and Martin J. Donnelly performed all the analysis presented in this publication; Lilian Namuli, Erin Knight, Samuel Gonahasa, Amy Lynd, Ambrose Oruni, Catherine Maiteki‐Sebuguzi, Jimmy Opigo, Adoke Yeka, Agaba Katureebe, Mary Kyohere, Moses R. Kamya and Sarah G. Staedke conducted trials, entomological collections and laboratory processing; Harun N. Njoroge, Eric R. Lucas and Martin J. Donnelly wrote the manuscript with input from all authors; Grant Dorsey, Janet Hemingway, Sarah G. Staedke, Eric R. Lucas and Martin J. Donnelly supervised the study; all authors approved the final version of the manuscript.

## Funding

Research reported in this publication was supported by the National Institute of Allergy and Infectious Diseases of the National Institutes of Health under Award Number R01AI116811. The content is solely the responsibility of the authors and does not necessarily represent the official views of the National Institutes of Health. Harun Njoroge Ng'ang'a is supported by an MRC studentship (MR/R015678/1). Martin James Donnelly was supported by a Royal Society Wolfson Fellowship (RSWF\FT\180,003). The MalariaGEN Vector Observatory is supported by funding from Wellcome (220,540/Z/20/A, ‘Wellcome Sanger Institute Quinquennial Review 2021–2026’) and the Gates Foundation (INV‐001927, INV‐068808). Ambrose Oruni was supported by Wellcome Trust MSc Fellowship in Public Health and Tropical Medicine (Oruni‐203,511/Z/16/Z).

## Conflicts of Interest

The authors declare no conflicts of interest.

## Supporting information


**Figure S1:** Definition of ΔH12 peak regions and haplotype clustering boundaries.

**Figure S2:** PCA and windowed F_ST_ comparing Eastern vs. Western Uganda.

**Figure S3:** Msprime simulation results and power to detect population crashes under different N_e_ and sample sizes.

**Figure S4:** Genome‐wide SNP association results.

**Figure S5:** Power simulations for detecting allele‐frequency shifts.

**Figure S6:** Cyp6aa1‐Cyp6p2 locus details including duplication‐associated haplotypes.

**Figure S7:** Relationship between the 2La inversion background and the swept haplotype at ~34 Mb.

**Table S1:** Sample summaries including cluster‐ and round‐level mosquito catches during the LLINEUP trial and samples whose whole genome sequences were used in the study.

**Table S2:** Genetic regions where Eastern and Western Uganda *Anopheles gambiae* populations are significantly differentiated (F_ST_).

**Table S3:** Temporal changes in Anopheles density ratios during the LLINEUP trial.

**Table S4:** Population genetics analysis to infer changes in *An. gambiae* population size after Bed net distribution during the LLINEUP trial in Uganda.

**Table S5:** Haplotype cluster analysis evaluating genetic responses in *An. gambiae* to Bed Net Interventions.

**Table S6:** Top Single nucleotide polymorphism (SNPs) associated with main haplotypes in genomic regions where significant changes occurred during the LLINEUP bed net trial in Uganda.

**Table S7:** Changes in Knockdown resistance (KDR) haplotypes during the LLINEUP bednet intervention in Uganda.

## Data Availability

Epidemiological data from the LLINEUP cluster‐randomised trial are available via ClinEpiDB (LLINEUP Cluster Randomised Trial). Whole‐genome variant data (SNPs and CNVs) analysed in this study are available through the MalariaGEN *Anopheles gambiae* 1000 Genomes Project (Ag1000G) and can be accessed using the malariagen‐data Python package or downloaded from the MalariaGEN (https://malariagen.github.io/vector‐data/ag3/download.html). The Ag1000G sample sets used were [“1288‐VO‐UG‐DONNELLY‐VMF00168”, “1288‐VO‐UG‐DONNELLY‐VMF00219”]. The whole genome sequences can also be accessed through the European Nucleotide Archive (Supporting Data 1). All analysis code (data processing, population genetic analyses, and figure generation) is available in a public GitHub repository: https://github.com/Harunnjoroge/llineup_publication.
