# Supplementary material for: Genetic Surveillance Reveals Differential Evolutionary Dynamic of Anopheles gambiae Under Contrasting Insecticidal Tools Used in Malaria Control
Source: Mol Ecol. 2026 Mar 3;35(5):e70284. doi: 10.1111/mec.70284 (PMC12954828; doi:10.1111/mec.70284)
Supplement: Supplementary file 2 — Figure S2: PCA and windowed FST comparing Eastern vs. Western Uganda. [file MEC-35-e70284-s002.pdf]

Genetic Surveillance Reveals Differential Evolutionary Dynamic of *Anopheles gambiae* Under Contrasting Insecticidal Tools used in Malaria control

Supplementary figure 2

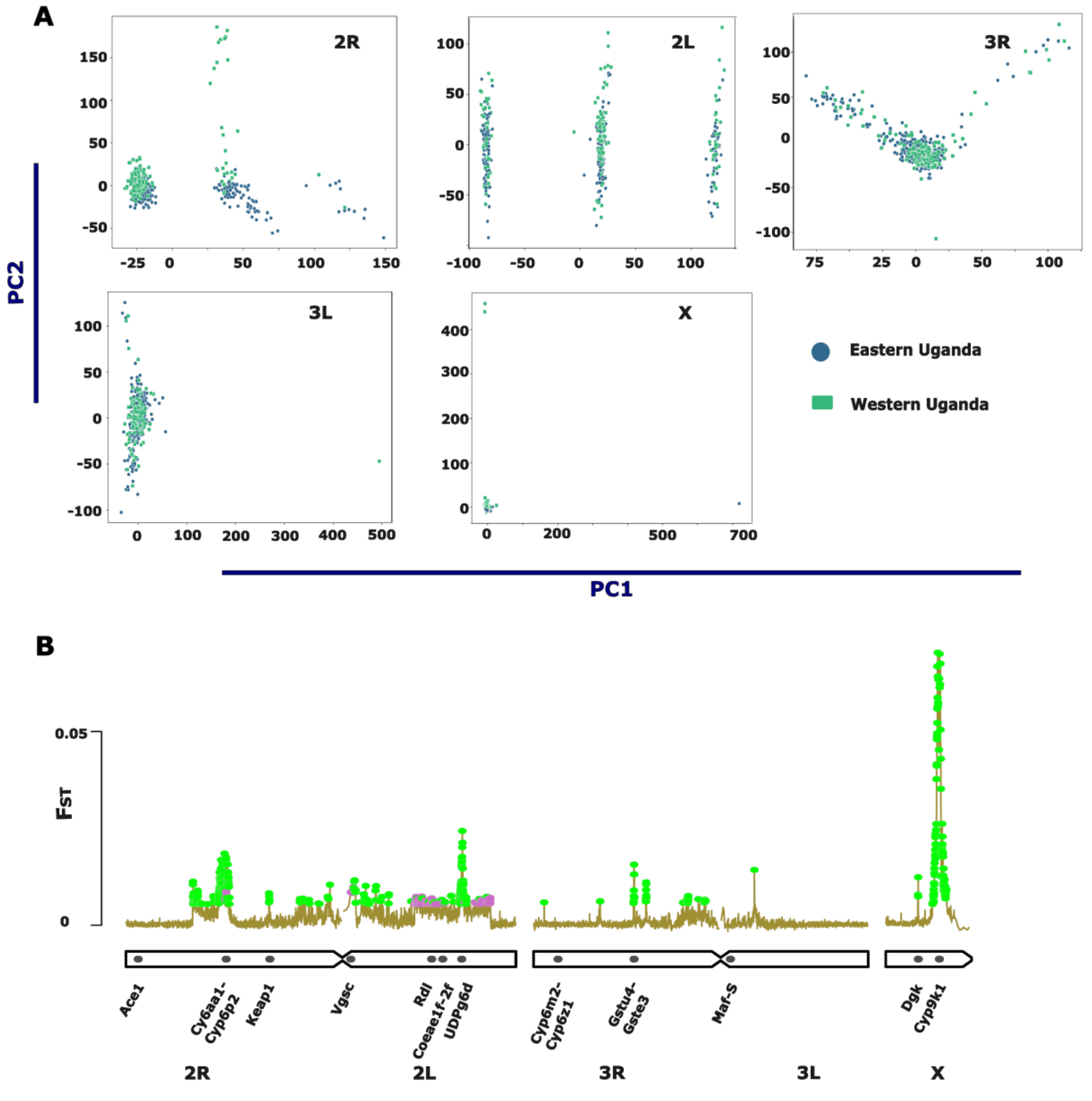

Supplementary Fig.2 Population structure of Anopheles gambiae population in Uganda.

***A:** Principal component analysis of An. gambiae population structure before net distribution based on chromosomes 2R, 2L, 3R, 3L and X did not reveal population structure between mosquitoes collected in Eastern vs Western Uganda but structured in chromosome 2R and 2L based on chromosomal inversions (2Rb and 2La respectively). **B:** Genome wide windowed F_{ST} analysis revealed population differentiation between the two populations mostly in loci associated with insecticide resistance. On the F_{ST} plot, colored dots mark windows surpassing the permutation-based significance threshold (green: significant; purple: marginal).*
