# Supplementary material for: Genetic Surveillance Reveals Differential Evolutionary Dynamic of Anopheles gambiae Under Contrasting Insecticidal Tools Used in Malaria Control
Source: Mol Ecol. 2026 Mar 3;35(5):e70284. doi: 10.1111/mec.70284 (PMC12954828; doi:10.1111/mec.70284)
Supplement: Supplementary file 3 — Figure S3: Msprime simulation results and power to detect population crashes under different Ne and sample sizes. [file MEC-35-e70284-s011.pdf]

Genetic Surveillance Reveals Differential Evolutionary Dynamic of *Anopheles gambiae* Under Contrasting Insecticidal Tools used in Malaria control

Supplementary Figure 3

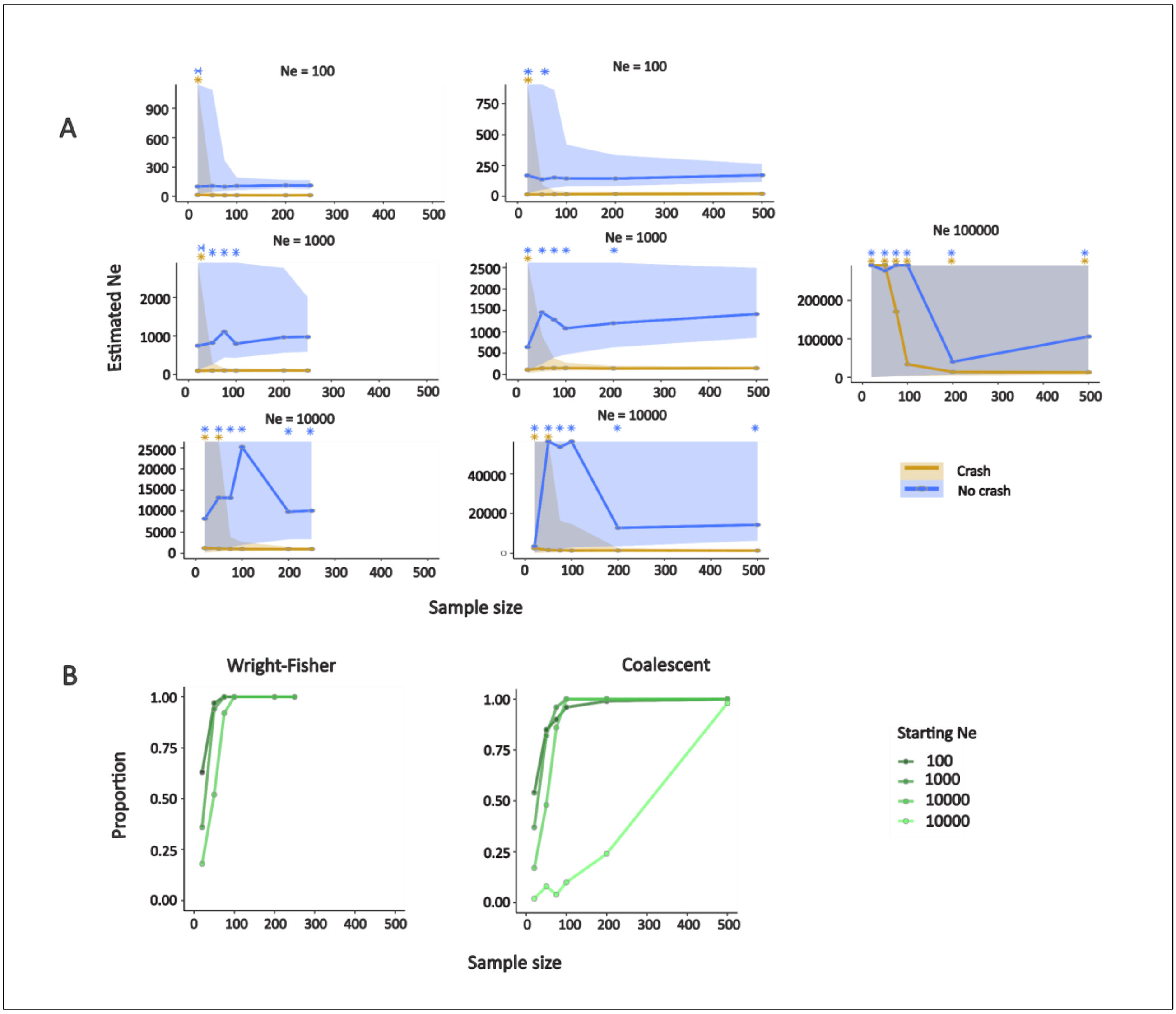

Supplementary Fig.3. Sample Size Requirements for LD-Based N_e Estimation and Detection of Population Decline

***Panel A**: shows the median N_e estimated from the simulations, along with the 2.5% and 97.5% centile, showing results from the Wright-Fisher simulations on the left, and coalescent simulations on the right, at a range of sample sizes (x axis). For Wright-Fisher simulations, only 250 samples were simulated, and thus sample sizes above this could not be explored. Asterisks above the plot at each point indicates the 97.5% centile was infinite (i.e.: more than 2.5% of simulations resulted in an infinite estimate of N_e). Note that y axis ranges are not standardised. At a starting N_e of 100000, both crash and non-crash scenarios had infinite 97.5% centiles.*

***Panel B:** shows the proportion of simulations (out of 100, or 50 for simulations at N_e = 100000) in which a significant difference in r^2 was found between crash and no-crash populations. Already at a population size of 100000, a sample size of 200 in each population was insufficient to achieve good power, while a sample size of 500 (i.e.: 1000 samples total) was significant in 49 out of 50 simulations. However, given that An. gambiae typically has a population size in the millions (The Anopheles gambiae 1000 Genomes Consortium, 2017)a likely extrapolation of this is that a 10-fold crash would not be detectable in a natural population with this method.*
