# Supplementary material for: Genetic Surveillance Reveals Differential Evolutionary Dynamic of Anopheles gambiae Under Contrasting Insecticidal Tools Used in Malaria Control
Source: Mol Ecol. 2026 Mar 3;35(5):e70284. doi: 10.1111/mec.70284 (PMC12954828; doi:10.1111/mec.70284)
Supplement: Supplementary file 4 — Figure S4: Genome‐wide SNP association results. [file MEC-35-e70284-s009.pdf]

Genetic Surveillance Reveals Differential Evolutionary Dynamic of *Anopheles gambiae* Under Contrasting Insecticidal Tools used in Malaria control

Supplementary figure 4

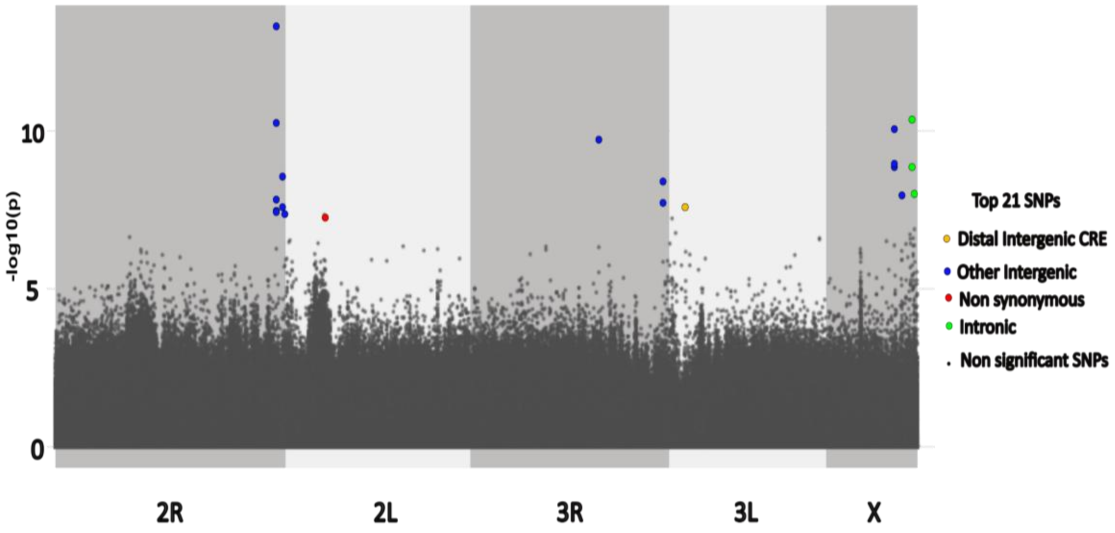

Supplementary Fig.4 A genome wide analysis of temporal variation in SNP frequency in Anopheles gambiae population driven by either pyrethroid exposure in standard pyrethroid only nets or Pyrethroid-PBO nets distributed in Uganda. *The highlighted P values (coloured) are SNPs that changed significantly during the intervention (FDR <0.05). One SNP in a red (2L:10334049, A>C, V390G) in AGAP005127 (RNA-binding protein 15) was non-synonymous, while three were intronic SNPs (in green) within CYP4G16 (X:22941281 and X:22941291) and Fatty acyl-CoA reductase (X:23560372). In Orange is 3L:4240178 which mapped to a distal intergenic cis regulatory element (CRE) 4.5kb upstream of AGAP010481 (solute carrier 6 transporter) gene. The remaining SNPs in blue were intergenic SNPs which didn't map to any CRE based on the ATAC peaks data from (Ruiz et al., 2021).*
