# Supplementary material for: Genetic Surveillance Reveals Differential Evolutionary Dynamic of Anopheles gambiae Under Contrasting Insecticidal Tools Used in Malaria Control
Source: Mol Ecol. 2026 Mar 3;35(5):e70284. doi: 10.1111/mec.70284 (PMC12954828; doi:10.1111/mec.70284)
Supplement: Supplementary file 5 — Figure S5: Power simulations for detecting allele‐frequency shifts. [file MEC-35-e70284-s014.pdf]

Genetic Surveillance Reveals Differential Evolutionary Dynamic of *Anopheles gambiae* Under Contrasting Insecticidal Tools used in Malaria control

Supplementary figure 5

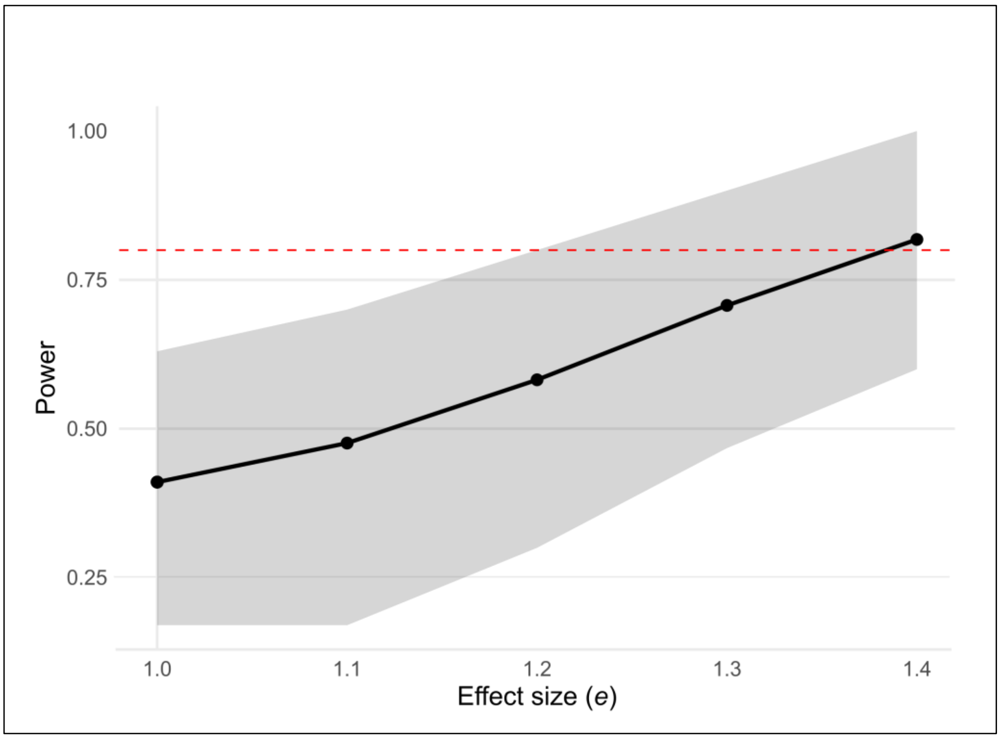

Supplementary Fig 5. Power simulations for detecting adaptive allele frequency shifts. *The dotted red line is the 80% power threshold in which with sample size (185 in baseline and 53 in post-intervention) we can detect a 29% change in SNP frequency. With these sample sizes, our power to detect small changes was limited.*
