# Supplementary material for: Genetic Surveillance Reveals Differential Evolutionary Dynamic of Anopheles gambiae Under Contrasting Insecticidal Tools Used in Malaria Control
Source: Mol Ecol. 2026 Mar 3;35(5):e70284. doi: 10.1111/mec.70284 (PMC12954828; doi:10.1111/mec.70284)
Supplement: Supplementary file 6 — Figure S6: Cyp6aa1‐Cyp6p2 locus details including duplication‐associated haplotypes. [file MEC-35-e70284-s013.pdf]

Genetic Surveillance Reveals Differential Evolutionary Dynamic of *Anopheles gambiae* Under Contrasting Insecticidal Tools used in Malaria control

Supplementary figure 6

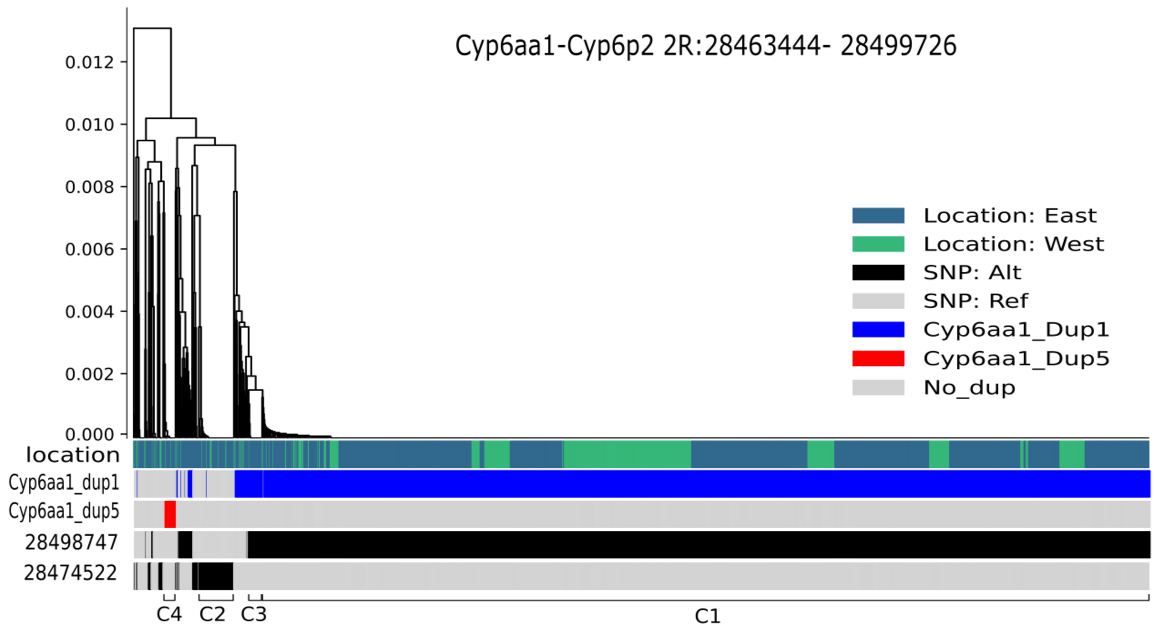

Supplementary Fig.6 Haplotype dendrogram of the Cyp6aa1-Cyp6p2 region showing the main haplotype is associated with Cyp6aa1 duplication. *Each dendrogram leaf represents a haplotype. Coloured bars beneath each dendrogram show for each haplotype it's geographical origin (green = Western Uganda, navy blue = Eastern Uganda) and the presence (blue / red) or absence (light grey) of known CNV alleles on that haplotype. SNPs tagging the haplotype clusters are represented in black colour while reference allele is light grey.*
