# Supplementary material for: Genetic Surveillance Reveals Differential Evolutionary Dynamic of Anopheles gambiae Under Contrasting Insecticidal Tools Used in Malaria Control
Source: Mol Ecol. 2026 Mar 3;35(5):e70284. doi: 10.1111/mec.70284 (PMC12954828; doi:10.1111/mec.70284)
Supplement: Supplementary file 7 — Figure S7: Relationship between the 2La inversion background and the swept haplotype at ~34 Mb. [file MEC-35-e70284-s005.pdf]

Genetic Surveillance Reveals Differential Evolutionary Dynamic of *Anopheles gambiae* Under Contrasting Insecticidal Tools used in Malaria control

Supplementary figure 7

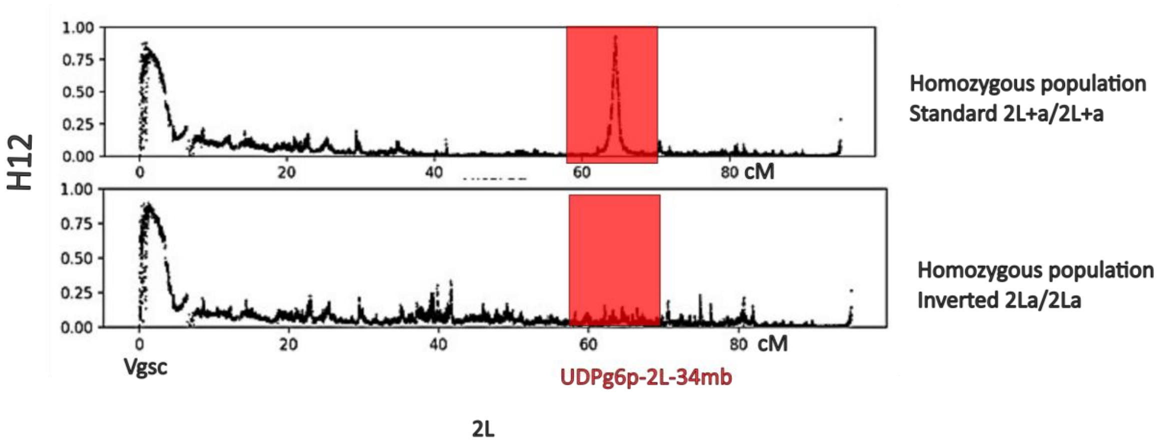

Supplementary Fig.7. Selective Sweep at ~34 Mb Restricted to mosquito population carrying the Standard (2L+a) chromosomal form
