# Supplementary material for: Genetic Surveillance Reveals Differential Evolutionary Dynamic of Anopheles gambiae Under Contrasting Insecticidal Tools Used in Malaria Control
Source: Mol Ecol. 2026 Mar 3;35(5):e70284. doi: 10.1111/mec.70284 (PMC12954828; doi:10.1111/mec.70284)
Supplement: Supplementary file 8 — Table S1: Sample summaries including cluster‐ and round‐level mosquito catches during the LLINEUP trial and samples whose whole genome sequences were used in the study. [file MEC-35-e70284-s012.pdf]

Genetic Surveillance Reveals Differential Evolutionary Dynamic of *Anopheles gambiae* Under Contrasting Insecticidal Tools used in Malaria control

Supplementary Table 1

Supplementary Table 1a. Total number of female mosquitoes collected from 104 health sub districts across all 5 collection rounds In the LLINEUP bednet trial in Uganda. *Reproduced from (Lynd et al., 2019)*.

	Baseline/1	Round 2	Round 3	Round 4	Round 5[b]
An. gambiae s.s	1284	191	441	256	815
An. arabiensis	80	36	61	117	74
An. funestus	432	194	250	719	435
Other Anophelines[a]	1	2	3	1	3

[a] **other *Anophelines* were *An. coluzzii*, *An.leesoni*, *An. rivulorum* and *An. mouchette***

[b] ***Data from 90 HSD instead of 104 due to logistics issues arising from covid outbreak***.

Supplementary Table 1b. The number of samples used in the genomic analysis. *Because of the low number of intermediate collections across the 4 cohorts, only baseline and round 5 (25 months post intervention) were included in the analysis.*

Location	Trial arm	Baseline	Round 2	Round 3	Round 4	Round 5	Total
**East**	Nonpbo	**132**	47	148	65	**192**	584
	Pbo	**112**	0	10	1	**39**	162
**West**	Nonpbo	**75**	3	6	61	**30**	175
	Pbo	**73**	1	4	0	**14**	92
