# Supplementary material for: Genetic Surveillance Reveals Differential Evolutionary Dynamic of Anopheles gambiae Under Contrasting Insecticidal Tools Used in Malaria Control
Source: Mol Ecol. 2026 Mar 3;35(5):e70284. doi: 10.1111/mec.70284 (PMC12954828; doi:10.1111/mec.70284)
Supplement: Supplementary file 9 — Table S2: Genetic regions where Eastern and Western Uganda Anopheles gambiae populations are significantly differentiated (FST). [file MEC-35-e70284-s006.pdf]

Genetic Surveillance Reveals Differential Evolutionary Dynamic of *Anopheles gambiae* Under Contrasting Insecticidal Tools used in Malaria control

Supplementary Table 2. Genetic regions where Eastern and Western Uganda Anopheles gambiae populations are significantly differentiated (F_{ST})

REGION	GENOMIC POSITION
**2R**	
**2RB INVERSION**	19045864-29408339
**KEAP1**	40718771-40825012
**PRE CENTROMERE REGION**	49400062-58015826
**2L**	
**VGSC AND POST-VGSC REGION**	3254470-3800315
**2LA INVERSION**	5508554-35310429
**3R**	
**PEAK1**	3010777(midpoint ±500)
**PEAK 2**	18932708-18951455
**GSTU4-GSTE3**	28455408-28653746
**PEAK 3**	31955614-32079510
**PRE-CENTROMERE PEAKS**	42524467-48905895
**3L**	
**PEAK 1**	9611887(midpoint ±500)
**X**	
**DIACYLGLYCEROL KINASE**	9154659-9225697
**CYP9K1**	13283325-17213545
