# Supplementary material for: Genetic Surveillance Reveals Differential Evolutionary Dynamic of Anopheles gambiae Under Contrasting Insecticidal Tools Used in Malaria Control
Source: Mol Ecol. 2026 Mar 3;35(5):e70284. doi: 10.1111/mec.70284 (PMC12954828; doi:10.1111/mec.70284)
Supplement: Supplementary file 10 — Table S3: Temporal changes in Anopheles density ratios during the LLINEUP trial. [file MEC-35-e70284-s008.pdf]

**Genetic Surveillance Reveals Differential Evolutionary Dynamic of *Anopheles gambiae* Under Contrasting Insecticidal Tools used in Malaria control**

***Supplementary Table 3. Anopheles density ratios over time after LLIN distribution***

| **ROUND** | **DENSITY RATIO PBO BEDNETS** | **DENSITY RATIO STANDARD BEDNETS** | **EFFECT SIZE*** | **P VALUES*** |
|---|---|---|---|---|
| BASELINE | Reference | Ref. | Ref. | Ref. |
| 6 MONTHS | 0.2129103 | 0.8683859 | -0.6221 | **0.000326** |
| 12 MONTHS | 0.3625230 | 1.0792079 | -0.4677 | **0.000539** |
| 18 MONTHS | 0.4802311 | 1.3610011 | -0.2093 | 0.27 |
| 25 MONTHS | 0.6924962 | 1.7537129 | -0.1156 | 0.36 |

**The effect size and p value were calculated using a generalised linear mixed model in which the net type and round were fixed effects while household id was fitted as a random effect.*
