# Supplementary material for: Genetic Surveillance Reveals Differential Evolutionary Dynamic of Anopheles gambiae Under Contrasting Insecticidal Tools Used in Malaria Control
Source: Mol Ecol. 2026 Mar 3;35(5):e70284. doi: 10.1111/mec.70284 (PMC12954828; doi:10.1111/mec.70284)
Supplement: Supplementary file 11 — Table S4: Population genetics analysis to infer changes in An. gambiae population size after Bed net distribution during the LLINEUP trial in Uganda. [file MEC-35-e70284-s001.pdf]

Genetic Surveillance Reveals Differential Evolutionary Dynamic of *Anopheles gambiae* Under Contrasting Insecticidal Tools used in Malaria control

Supplementary Table.4 Population genetics analysis to infer changes in An. gambiae population size after Bed net distribution during the LLINEUP trial in Uganda

Region	Cohort	LD[a]	N_e[b]	Nucleotide diversity (π)[c]
All	All baseline	0.0007[0.00005, 0.015]	Inf [3636, Inf]	0.0214 [0.0201, 0.0226]
	All post	0.0007[0.00005, 0.015]	Inf [4036, Inf]	0.0211 [0.0199, 0.0224]
	PBO baseline	0.007[0.0003, 0.08]	3348 [1273, Inf]	0.0212 [0.0199, 0.0224]
	PBO post	0.007[0.0003, 0.08]	Inf [1554, Inf]	0.0211 [0.0199, 0.0224]
	Standard baseline	0.001[0.0001, 0.02]	7210 [3169, Inf]	0.0215 [0.0202, 0.0227]
	Standard post	0.001[0.0001, 0.02]	Inf [3478, Inf]	0.0211 [0.0199, 0.0224]
West	PBO baseline	0.051[1.7e-32, 0.412]	1008 [229, Inf]	0.0212 [0.020, 0.0225]
	PBO post	0.051[2.3e-32, 0.412]	Inf [244, Inf]	0.0210 [0.0197, 0.0223]
	Standard baseline	0.017[0.00009, 0.209]	3408 [708, Inf]	0.0211 [0.0199, 0.0224]
	Standard post	0.017[0.00009, 0.210]	Inf [702, inf]	0.0211 [0.0199, 0.0224]
East	PBO baseline	0.011[0.00008, 0.147]	949 [853, Inf]	0.0212 [0.0199, 0.0224]
	PBO post	0.011[0.00008, 0.149]	12876 [667, Inf]	0.0211 [0.0199, 0.0224]
	Standard baseline	0.002[0.00004, 0.053]	Inf [2429, Inf]	0.0211 [0.0199, 0.0224]
	Standard post	0.002[0.00004, 0.053]	Inf [2176, Inf]	0.0211 [0.0199, 0.0224]

[a] LD is the median of R squares calculated from squared correlation coefficient (r^2) for SNPs pairs within chromosomes 2 vs 3. In brackets are the 5 and 95% percentiles. [b]N_e is an estimate from LD and in brackets are 95% CI estimated from 1000 replicates. [c]π is the nucleotide diversity estimate and in brackets 95% CI.
