# Supplementary material for: Genetic Surveillance Reveals Differential Evolutionary Dynamic of Anopheles gambiae Under Contrasting Insecticidal Tools Used in Malaria Control
Source: Mol Ecol. 2026 Mar 3;35(5):e70284. doi: 10.1111/mec.70284 (PMC12954828; doi:10.1111/mec.70284)
Supplement: Supplementary file 12 — Table S5: Haplotype cluster analysis evaluating genetic responses in An. gambiae to Bed Net Interventions. [file MEC-35-e70284-s010.pdf]

Genetic Surveillance Reveals Differential Evolutionary Dynamic of *Anopheles gambiae* Under Contrasting Insecticidal Tools used in Malaria control

Supplementary Table 5. Haplotype Cluster Analysis Highlights Region- and Net-Specific Genetic Responses in Anopheles gambiae to Bed Net Interventions.

Region	Hypothesis	C1		C2		C3		C4	
		p	coef	p	coef	p	coef	p	coef
Cyp6aa1-Cyp6p2 (2R:28463444–28499726)	H_3[a]	**0.02**	0.4	**0.002 [0.02]**	-1.02	0.28	0.58	0.98	-0.013
	H_4[b]-PBO	0.064	0.7	**0.0022 [0.02]**	-48	**0.03**	2.3	0.8	0.32
	H_4[b]-standard	0.12	0.31	**0.03**	-0.78	0.93	-0.055	0.94	-0.043
Vgsc (2L:2791320–2893275)	H_3	0.3	-0.12	0.38	-0.13	0.08	0.68	**0.012**	1.3
	H_3[c]-East	0.37	-0.12	0.51	0.09	0.08	0.9	0.36	1.1
	H_3[c]- West	0.79	-0.07	**0.007**	-0.8	0.69	0.22	**0.02**	1.3
	H_4- PBO	0.063	-0.42	0.91	0.026	0.36	0.6	0.13	1.1
	H_4-standard	0.92	0.015	0.1	-0.29	0.11	0.77	**0.029**	1.8
2L-34mb (2L:34081017–34101131)	H_3	0.35	0.16	0.65	0.12	0.074	-0.86	0.47	0.51
	H_4-PBO	**0.0003 [0.003]**	0.9	0.54	-0.31	0.89	0.15	0.34	-19
	H_4-standard	0.7	-0.08	0.49	0.21	**0.039**	-1.1	0.12	1.4
Dgk (X:9179019–9185374)	H_3	**0.0004 [0.003]**	0.87	0.83	-0.05	0.053	-0.96	0.69	0.28
	H_4-PBO	0.42	0.37	0.94	0.031	0.75	-0.25	0.32	-20
	H_4-standard	**2.7e-05 [0.0005]**	1.1	0.69	-0.098	**0.024**	-1.4	0.63	0.4
Cyp9k1 (15216225–15271654)	H_3	0.22	0.18	0.91	-0.03	0.95	-0.02	0.11	-0.84
	H_4-PBO	0.29	-0.32	0.4	0.43	0.53	-0.38	0.88	0.13
	H_4-standard	**0.0089 [0.04]**	0.44	0.34	-0.24	0.49	0.34	0.063	1.4

In bold are raw p values of haplotypes that changed significantly (<0.05) but after FDR correction only those with the square brackets were below the set significance threshold (5%).

[a] *model used to test H_3: variant ~ Round (1 vs 5) + location (east & west) + llin (PBO & standard) + 1|health subdistrict*

[b] *model used to test H_4[b]: variant ~ Round (1 vs 5) + location (east & west) + 1|health subdistrict (Data split by net type)*

[c] *model used to test H_4[c]: variant ~ Round (1 vs 5) + llin (PBO & standard) + 1|health subdistrict (Data split by location)*
