# Supplementary material for: Genetic Surveillance Reveals Differential Evolutionary Dynamic of Anopheles gambiae Under Contrasting Insecticidal Tools Used in Malaria Control
Source: Mol Ecol. 2026 Mar 3;35(5):e70284. doi: 10.1111/mec.70284 (PMC12954828; doi:10.1111/mec.70284)
Supplement: Supplementary file 13 — Table S6: Top Single nucleotide polymorphism (SNPs) associated with main haplotypes in genomic regions where significant changes occurred during the LLINEUP bed net trial in Uganda. [file MEC-35-e70284-s003.pdf]

Genetic Surveillance Reveals Differential Evolutionary Dynamic of *Anopheles gambiae* Under Contrasting Insecticidal Tools used in Malaria control

Supplementary Table 6. Top Single nucleotide polymorphism (SNPs) associated with main haplotypes in genomic regions where significant changes occurred during the LLINEUP bed net trial in Uganda

**2R:28463444–28499726 (*Cyp6aa1-Cyp6p2*)**	2R:28500104<br>2R:28499987<br>2R:28499354<br>2R:28498747<br>2R: 28497958	2R:28476019<br>2R:28475974<br>2R:28474555<br>2R:28474522<br>2R:28507610
**2L:2791320–2893275 (*Vgsc*)**	2L:2883338<br>2L:2569930<br>2L:2300012<br>2L:2300046	2L:2409947<br>2L:2380726<br>2L:2383037<br>2L:2321129
**2L:34081017–34101131 (*2L-34mb*)**	2L:34076000<br><br>2L: 34073954<br>2L:34093806<br>2L:34100535<br>2L:34103278	2L:34100533<br><br>2L:34098712<br>2L:34097024<br>2L:34098608<br>2L:34096945
**X:9179019–9185374 (*Dgk*)**	X:9179818<br><br>X:9183213<br>X:9183402<br>X:9190710	X:9185342<br><br>X:9183189<br>X:9181789<br>X:9181138
**X:15216225–15271654 (*Cyp9k1*)**	X:15239300<br>X:15238600<br>X:15237378<br>X:15236958<br>X:15236122	X:15247409<br>X:15247243<br>X:15242464<br>X:15238589<br>15206248
