# Supplementary material for: Genetic Surveillance Reveals Differential Evolutionary Dynamic of Anopheles gambiae Under Contrasting Insecticidal Tools Used in Malaria Control
Source: Mol Ecol. 2026 Mar 3;35(5):e70284. doi: 10.1111/mec.70284 (PMC12954828; doi:10.1111/mec.70284)
Supplement: Supplementary file 14 — Table S7: Changes in Knockdown resistance (KDR) haplotypes during the LLINEUP bednet intervention in Uganda. [file MEC-35-e70284-s007.pdf]

Genetic Surveillance Reveals Differential Evolutionary Dynamic of *Anopheles gambiae* Under Contrasting Insecticidal Tools used in Malaria control

Supplementary Table 7. Change in Knockdown resistance (KDR) haplotypes during the LLINEUP bednet intervention in Uganda.

Kdr haplotypes	***H3***		***H3_east***		***H3_west***		***H4 PBO***		***H4 non-PBO***	
	p	coef	p	coef	p	coef	p	coef	p	coef
S1	0.17	-0.2	0.47	-0.1	0.2	-0.3	**0.04 [0.3]**	-0.5	0.86	-0.03
S3	0.28	-0.2	0.9	0.01	0.05 [0.3]	-0.6	0.65	-0.1	0.15	-0.3
S4	-	-	-	-	-	-	-	-	1	-6.3
S	0.77	0.2	0.7	-0.4	0.33	1	0.12	1.3	0.33	-1.3
F3	0.22	1.3	-	-	0.22	1.3	**0.007 [0.049]**	33	-	-
F5	**0.006 [0.04]**	1.8	0.09	21	**0.01 [0.08]**	1.6	**0.04 [0.3]**	1.8	**0.02 [0.1]**	3
F	0.33	0.6	0.43	-1.8	0.28	0.7	0.5	0.6	0.62	0.5
WT	0.4	-0.5	0.18	-1	0.4	0.8	0.34	-0.7	0.64	-0.4

*We tested two hypotheses: H3 -The pyrethroid in both net types will drive similar changes in KDR haplotypes frequencies. This was tested in both regions combined or separately. H4- Net specific changes in KDR haplotype frequencies will be observed. The p values in bold are where the haplotype change was significant (in square brackets) after correction for multiple testing (Bonferroni = 5%)
